# Oxytocin as a treatment for high-risk psychosis or early stages of psychosis: a mini review

**DOI:** 10.3389/fpsyt.2023.1232776

**Published:** 2023-08-17

**Authors:** Stavroula I. Bargiota, Anna V. Papakonstantinou, Nikolaos G. Christodoulou

**Affiliations:** ^1^Department of Psychiatry, Faculty of Medicine, University of Thessaly, Larissa, Greece; ^2^Faculty of Health Sciences, School of Medicine, Aristotle University of Thessaloniki, Thessaloniki, Greece

**Keywords:** early stages, high-risk, oxytocin, psychosis, treatment

## Abstract

Individuals at clinical high risk for psychosis (CHR-P) present as help-seeking individuals with social deficits as well as cognitive and functional impairment and have a 23–36% risk of transition to first-episode psychosis. The therapeutic role of intranasal oxytocin (ΟΤ) in psychiatric disorders has been widely studied during the last decades, concerning its effects on social behavior in humans. A literature search was conducted via Pubmed and Scopus, using the search terms “oxytocin” and “psychosis.” Six studies were included in the current review. There were differences in terms of demographics, intervention type, and outcome measures. ΟΤ may affect the social cognition skills of people at prodromal and early stages of psychosis, but its effect on clinical symptoms is ambiguous. Because of the high level of heterogeneity of existing studies, more original studies are needed to examine and clarify whether OT improves high-risk and early psychosis populations.

## 1. Introduction

Schizophrenia usually starts during late adolescence and early adulthood affecting approximately 1% of the general population (1). This onset of psychosis is often preceded by attenuated psychotic symptoms which represent a significantly high risk of developing psychotic disorders (2). Individuals at clinical high risk for psychosis (CHR-P) (3) present as help-seeking individuals with social deficits and cognitive and functional impairment. They also have a 23–36% risk of transition to first-episode psychosis (FEP) after a 3-year follow-up (4). Important neurodevelopmental changes take place during late adolescence and early adulthood, which renders this developmental stage amenable to therapeutic and preventive interventions. Early psychosis is mainly treated with pharmacological and psychological interventions (5). According to the current literature, novel treatments are needed, capable of preventing psychosis, reducing the severity of the symptoms, and ameliorating social and functional impairment (6).

The neuro-hypophyseal hormone, nine amino-acid peptide oxytocin (OT) is produced in the hypothalamus and is secreted into the bloodstream by the posterior pituitary gland. Α strong increase in the density of myometrial OT receptors, during early labor and the stimulation of nipples during breastfeeding (7) promotes the OT release into the bloodstream. ΟΤ mediates various processes in the brain and acts, overall, as a neurotransmitter (8), as shown early in animal research (originating from Isel and Young), (9) thus producing findings for replication in human studies.

A single dose of intranasal OT, ranging between 10 and 40 International Units (IU), is well tolerated, increases the answering score in various social cognition tasks (10) and tasks investigating empathy, deception, and sarcasm, and ameliorates facial emotion recognition in people diagnosed with schizophrenia (11).

### 1.1. Clinical high-risk for psychosis (CHR-P) and early stages of psychosis individuals and possible intervention targets

To prevent psychosis, the focus is concentrated on the detection and improvement of symptoms in individuals at clinical high risk for psychosis (CHR-P) and people in the early stages of psychosis (6). Several studies have shown that impaired social functioning at the prodromal stage plays an important role in predicting the transition to full-blown psychosis (12). In addition, no pharmacological agents are licensed to treat the people at CHR-P, because there are not any practicable interventions targeting the reduction of attenuated positive psychotic symptoms in this population (6). Concerning the early stages of psychosis, patients usually deteriorate within the first 5 years, especially if left untreated. Nevertheless, medication treats positive symptoms, but does not improve social functioning or negative symptoms in early psychosis (5).

The oxytocinergic system is a key modulator of social cognition and behavior, representing a promising intervention for improvement of social dysfunction, also in CHR-P subjects. OT is currently being investigated as a potential treatment for other neuropsychiatric disorders associated with social impairment, such as schizophrenia and autism spectrum disorder, given its ability to interact with the central dopamine systems (13). Still, in contrast to other disorders, studies investigating the role of intranasal OT in ameliorating symptoms of CHR-P individuals or at early stages of psychosis are few (14).

The studied population consists of CHR-P who present with prodromal symptoms of psychosis and “early psychosis” patients not sufficiently regulated with their present anti-psychotic treatment. The intervention studied was the intranasal administration of oxytocin. Comparison with healthy controls was not necessary in the included studies, and the outcome was defined by the alteration of psychotic symptoms. This paper aims to review the current published evidence on the effects of OT on individuals at CHR-P or with early symptoms of psychosis.

## 2. Materials and methods

### 2.1. Data sources and search terms

The literature search was conducted in January 2023 via Pubmed and Scopus, using Boolean operators to combine the search terms “oxytocin” and “psychosis” (“oxytocin” AND “psychosis”) published from 01/01/1982 until 20/01/2023, including abstracts, in English language only. The search was limited to human studies. We used these terms to improve our search and enable us to scan the literature manually, instead of using the terms oxytocin AND high-risk or ultra-high risk which resulted in very few studies.

### 2.2. Inclusion and exclusion criteria

The primary predetermined inclusion criteria were as follows: (1) randomized controlled trials (RCTs) relating OT with prodromal and recent psychosis; (2) participants were adults or adolescents that fulfilled the Ultra High Risk (UHR) for Psychosis criteria or were diagnosed with early psychosis without (a) current substance dependence on alcohol or drugs (b) intellectual disability (intelligence quotient <70), (c) history of a significant neurological disorder, and (d) florid psychotic or related symptoms likely to require immediate intervention (e.g., suicidality); (3) the intervention used was the intranasal administration of OT; and (4) outcomes were the oxytocin-induced alterations to the different domains that each study examined.

We excluded abstracts, systematic reviews, meta-analyses, studies in healthy volunteers, non-human studies, studies for which there was insufficient data retrieval and non-English studies.

### 2.3. Study selection and data extraction

The next step required an electronic, manual search of the resulting articles and abstracts’ list. Articles and abstracts were scrutinized for relevancy, following PDF access and eligibility assessment (through full-text reading) by NC and SB. Disagreements were solved through consensus with AP.

Two independent researchers, SB and NC screened the titles and abstracts of all relevant publications as well as the references of each article to retrieve more sources. Full-text articles were obtained for all the studies considered compatible based on the abstract screening and then were further reviewed for eligibility.

**Figure 1:**
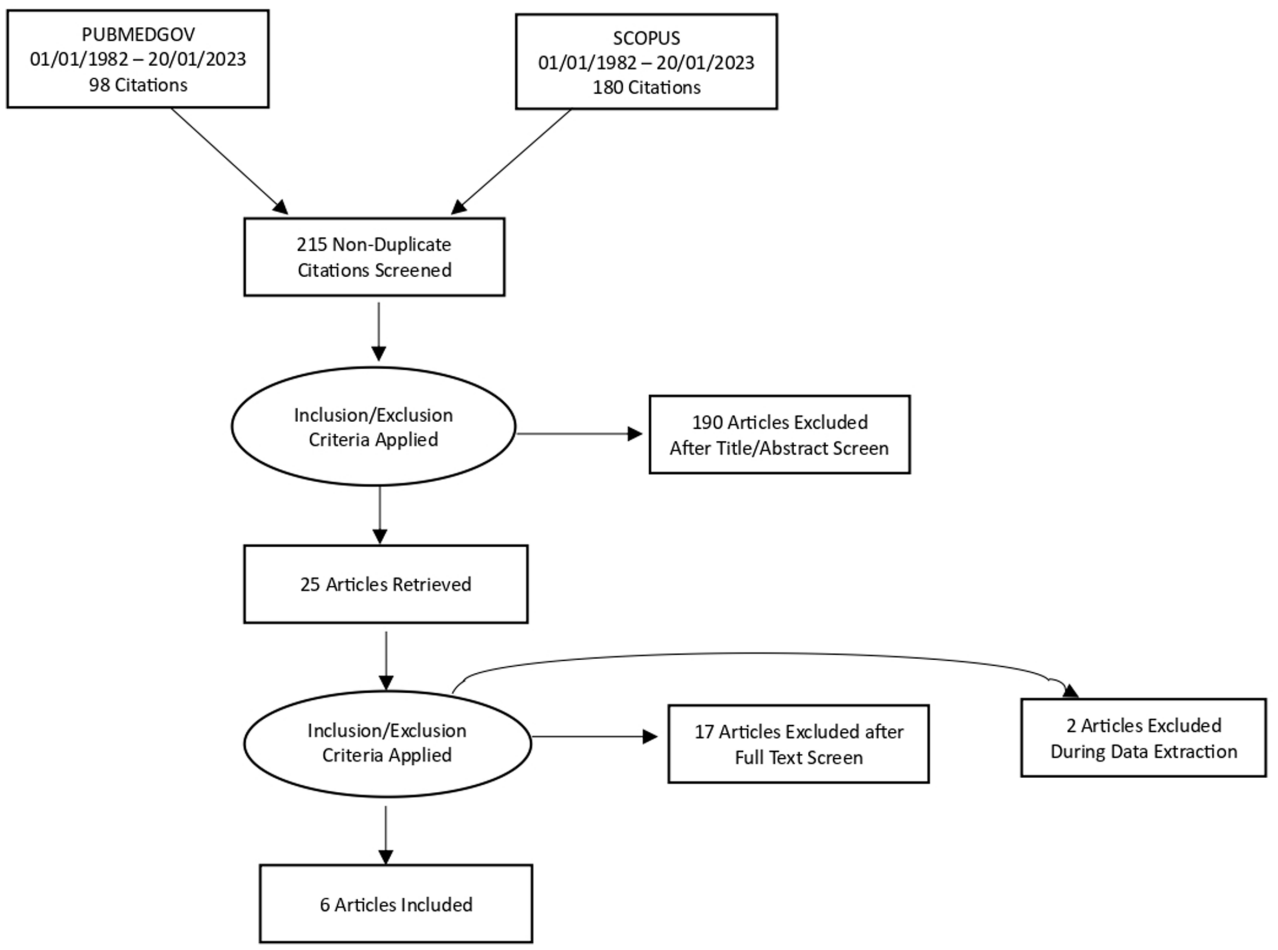
Study selection flowchart.

After the initial search, two hundred fifteen (215) non-duplicate English-language articles fulfilled the primary inclusion criteria. We excluded two hundred seven (207) articles in total, of which one hundred ninety (190) were excluded due to irrelevance and seventeen (17) articles because of the unacceptable level of disease, participants, or outcomes. Eight articles were further reviewed for eligibility. Two papers were excluded as they were non-interventional studies, and six articles were found eligible for the review. The study selection flow chart is shown in Figure 1.

## 3. Characteristics of the included studies

A summary of the six finally selected studies is shown in Table 1. The six studies included in the current review involved a total of 131 subjects (109 men and 22 women). Four studies used the same cohort of 30 subjects. There were differences between studies in terms of demographics, intervention type, and outcome measures. Five studies recruited only patients (15–19), whereas one study compared patients and healthy participants (14). The intervention was 40 IU of intranasal OT or placebo in five studies, whereas the intervention in the sixth included study by Cacciotti-Saija et al. (15) was 24 IU of intranasal OT or placebo and Social Cognition Therapy (SCT).

**Table 1:** Summary of studies relating the impact of oxytocin on individuals at Clinical High Risk for Psychosis (CHR-P) and Early Psychosis (EP).

Study (year)	Participants (number)	Age (years) Mean (SD)	Gender Male, %	Intervention (number receiving)	Control (number)	Outcome	Results
Davies et al. (15)	CHR-P* (30)	Cross-over design 23.2 (4.7)	100%	OT* 40 IU (28 for ACC* and thalamus) (26 hippocampus) Single dose	Placebo (28 for ACC* and thalamus) (26 hippocampus)	Neurochemical effects	No effects in metabolites Increase in choline
Davies et al. (21)	CHR-P* (30)	Crossover design 23.2 (4.7)	100%	OT* 40 IU (29) Single dose	Placebo (29)	Neurophysiological effects	Increase in resting cerebral perfusion in the hippocampus
Martins et al. (14)	CHR-P* (30) Healthy controls (17)	Crossover Design CHR-P* 22.93 (4.77) HC* 24.4 (5.33)	100%	ΟΤ* 40 IU (29 + 17) Single dose	Placebo (29 + 17)	Effects on autonomic regulation	Increase in cardio-parasympathetic activity
Schmidt et al. (22)	CHR-P* (30)	Crossover design 22.48 (4.68)	100%	OT* 40 IU (30) Single dose	Placebo (30)	Effects on brain activation during emotional or cognitive empathy Modulation of effects by baseline social–emotional abilities	Enhancement of neural efficiency in the inferior frontal gyrus in subjects with low baseline social–emotional abilities
Cacciotti et al. (20)	Early psychosis (≤3 years) (52)	Crossover design	69.2%	OT* 24 IU + SCT (27) Multi dose	Placebo + SCT* (25)	Effects on social cognition, social functioning, and clinical symptoms	No significant improvement in social cognition, symptom severity or social functioning
Dagani et al. (19)	Short-medium schizophrenia duration (<11 years) (32)	Crossover Design 30.4 (6.7)	81%	OT* 40 IU* + AP* treatment (16) Single dose	Placebo+AP* treatment (16)	Effects on psychotic symptoms	No significant benefits on clinical symptoms or psychosocial functioning

* CHR-P, Clinical High Risk for Psychosis; OT, Oxytocin; IU, International Units; ACC, Anterior Cingulate Cortex, HC: Healthy Controls; SCT, Social Cognition Training; AP treatment, Anti-Psychotic treatment.

The six studies selected were conducted in 3 countries worldwide, including the United Kingdom (14, 17–19); Australia (15) and Italy (16). The four United Kingdom studies examined various hypotheses using the same sample of 30 CHR-P males on two occasions, after intranasal oxytocin, and after placebo administration. In one study, the sample of 30 CHR-P males was investigated in relation to a sample of 17 healthy controls (14). The Australian study examined young people with early psychosis by administering OT or placebo. The Italian study also investigated young people with early psychosis, defining short-medium illness duration as <11 years.

### 3.1. Summary of evidence

For the assessment of the CHR-P state in the United Kingdom studies, the Comprehensive Assessment of At-Risk Mental States (CAARMS) 12/2006 criteria were used (20). Help-seeking, with functional decline individuals belong to the following subgroups of Ultra High-Risk criteria (a) attenuated psychotic symptoms, (b) brief limited intermittent psychotic symptoms (BLIPS), psychotic episode of duration <1 week, remitting without treatment, or (c) either schizotypal personality disorder or first-degree relative with a diagnosis of psychosis (20).

The first study tried to define the neurochemical effects of oxytocin in CHR-P subjects, since OT, as a neuropeptide, modulates glutamate neurotransmission in preclinical studies. Proton magnetic resonance spectroscopy (1H-MRS) was used to investigate the 30 CHR-P adult males once after 40 IU intranasal oxytocin and once after placebo. A single acute dose of OT did not alter glutamate or glutamate + glutamine in the left thalamus, anterior cingulate cortex (ACC), or left hippocampus approximately 75–99 min post-dosing. Moreover, OT administration did not affect the modulation of metabolites by OT, which differs in CHR-P subjects, like N-acetyl aspartate, myo-inositol, creatine, and choline in the hippocampus and thalamus, but resulted in a significant increase in the choline concentration in the ACC (17).

Afterward, the neurophysiological effects of OT in CHR-P individuals were examined (18). CHR-P subjects present with increased resting hippocampal blood flow (21), which results in clinical implications, like non-remission or transition to full-blown psychosis. A single dose of intranasal OT enhances social functioning and significantly affects resting hippocampal blood flow in healthy individuals. Moreover, it markedly increases resting cerebral perfusion in the left hippocampus, a region involved in the pathophysiology of the CHR-P state and the later onset of psychosis. In addition, the greatest changes are noticeable in the dentate gyrus and CA1 regions (22). In the Davies study, the whole-brain analysis revealed that the thalamus, parietal cortex, and cerebellum were also modulated by ΟΤ (18). Although OT can cause alterations, such as in brain regions taking part in the pathophysiology of psychosis onset, the results could illuminate its effects on symptoms, social cognition, functional decline, and other clinical presentations of the CHR-P state (18).

Subsequently, the United Kingdom studies’ authors used the same sample of 30 CHR-P males who had taken part in the two previous double-blinded, placebo-controlled, randomized, crossover MRI studies, to investigate the effects of intranasal oxytocin on heart rate (HR) and high-frequency HR variability (HF-HRV) between the 30 CHR-P subjects and 17 healthy men. People who present with impaired sympathetic or/and parasympathetic activity have been found to be more vulnerable to psychosis. The oxytocinergic system could contribute to the Autonomous Neural System (ANS) regulation, but its role at the beginning of psychosis is unclear (14).

Scientists combined a self-administered 40 IU dose of intranasal oxytocin with pulse plethysmography estimating HR and HRV after dosing as an index of cardio-parasympathetic activity in antipsychotic naïve CHR-P people, thus enabling the investigation without the adverse effects of antipsychotics on the ANS. In a proof-of-concept study, the placebo did not modulate resting HR or HF-HRV, neither in CHR-P, nor in healthy men. However, intranasal OT increased HF-HRV in CHR-P but not in healthy individuals. This highlights the necessity to examine the role of intranasal OT in regulating the ANS in CHR-P people, which is activated during stressful situations and its activation is linked to high cardiovascular and psychosis-triggering risk. Under these circumstances, intranasal OT can be a valuable agent in ameliorating parasympathetic activity to prevent the transition into psychosis (14).

The fourth United Kingdom study was the first fMRI study that explored the impact of an acute OT dose on brain activation during inferring others’ beliefs and social emotions in CHR-P subjects (19). CHR-P state is associated with decreased OT receptor methylation, which is related to the development of negative symptoms of schizophrenia, such as decreased sociality and increased anhedonia (23). This system represents a fertile exploration field, in terms of administering the neuropeptide OT to CHR-P individuals, given its significant role in social behavior and cognition. Thus, 30 CHR-P males were self-administered and examined after a dose of 40 IU intranasal OT and after the placebo. A test (Reading of the Mind in the Eyes Test) was used before the first scan to verify the ability of baseline social–emotional skills. Brain activation was assessed with a modified version of the Sally-Anne task while the subjects were inferring others’ beliefs and social emotions (19).

OT did not affect brain activity while inferring others’ beliefs (cognitive empathy). However, it did cause a decrease in the response of the inferior frontal gyrus during emotional empathy (inferring others’ social emotions). Furthermore, during social emotion inference, baseline social–emotional skills modulated a neural response in the left inferior frontal gyrus, following an OT dose. This impacted the association between brain activation and task performance. More specifically, CHR-P people with impaired social–emotional skills appeared to fulfill tasks with increased accuracy using emotional empathy, as their brain activation decreased in the left inferior frontal gyrus. In contrast, CHR-P subjects with high social–emotional abilities did not exhibit this association. Abnormal awareness of emotions is met throughout psychosis and usually predicts low functionality. In conclusion, this study provides an idea for new therapeutic choices for prodromal psychosis, targeting different neural regions of the brain.

The subsequent two studies included in the review examined people in the early stages of psychosis. Cacciotti and her colleagues in Australia (15) assessed the impact of 6 weeks, twice daily, nasal administration, 24 IU of OT or placebo, combined with targeted Social Cognition Training (SCT) in 52 early-psychosis patients. They examined the OT or placebo plus SCT effects on improvement of social cognition and social functioning in a double-blind, randomized, controlled trial. Patients were examined at initial screening, post-treatment, and 3 months after the intervention. This trial addressed four domains of social-cognitive impairment – emotional recognition, social perception, theory of mind, and attributional style. Results showed that the OT and SCT patient group reported fewer psychotic symptoms. However, symptoms’ impact, social cognition, or social functioning were not improved.

The study of Dagani et al. (16) examined the impact of intranasal OT as augmentation therapy in 32 schizophrenic patients with a mean age of 30.4 (+/− 6.7) years and an average disorder duration of 3.7 years. They were randomly allocated to receive 40 IU OT or placebo once daily for a period of 4 months. This intervention confirmed the previous findings of Cacciotti et al. (15) who claimed that the symptomatic effects of OT on subjects with younger mean age, shorter duration of illness, and longer exposure to intranasal OT may not be easily detected. This finding can be attributed to the decreased severity and persistence of psychotic symptoms in early psychosis (16).

## 4. Discussion

This review included RCTs that examined intranasal OT administration on CHR-P and early psychosis patients. CHR-P patients present with prodromal symptoms of psychosis whereas “early psychosis” patients in this review present with remaining psychotic symptoms and are under antipsychotic medication. We took these two distinct patient groups as one because we assumed that the “early-psychosis” patients do not sufficiently improve with their present antipsychotic treatment and thus they participated in the included studies.

An extensive review of patients with schizophrenia from Goh et al. showed that not all symptoms of psychosis (general, positive, and negative) are targeted with OT treatment, as the bibliography presents conflicting results in the dosing regimens of OT administration and the different OT effects in clinical symptoms (24). A recent systematic review by Sabe et al. including only 9 RCTs, found no meaningful effect on negative symptoms using intranasal OT with moderate or higher doses (>40–80 IU) (25). However, it presented a potential advantage for the positive symptoms’ improvement. Additionally, the review by Martins et al., which explored RCTs examining the effects of the daily-repeated intranasal OT application on psychosis symptoms, showed the responses’ heterogeneity to the negative schizophrenia symptoms, and that total psychopathology, positive and negative symptoms in schizophrenia-suffering individuals were neither improved nor worsened. OT may have a small effect on the patients’ general symptoms (shown only after removing one influential study from their analysis) (26), which is in accordance with the acknowledged action of OT in regulating neurocognitive procedures and functionality. Nonetheless, the weakness in Martins’s and Goh’s review is the inclusion of studies with samples limited to men (at least 70%) in their analysis and patients with established schizophrenia diagnoses under stabilized, anti-psychotic medication, and to whom added OT addressed their residual symptoms.

It is worth mentioning that the Cacciotti study showed that the OT and SCT group reported fewer psychotic symptoms and overall, some improvement. However, the RCT with schizophrenia and schizoaffective participants by Buchanan et al. showed that there were no meaningful changes in social functioning, positive and negative symptoms, and the participants’ beliefs (asocial or defeatist) according to the treatment received (27). This difference may exist because these patients received different OT doses (in Cacciotti’s study a 24 IU OT regimen, whereas in Buchanan’s study a 36 IU OT dose), or due to different disease states. Nonetheless, both studies agree that OT does not ameliorate symptom severity or social functioning in psychotic patients receiving OT and SCT.

Furthermore, the two longer term studies did not find benefits. This could be taken into consideration for future study designs since over time dosing of OT seems to be well tolerated even in older ages. Even though, its role remains controversial, and more research is needed (28, 29).

### 4.1. Limitations

This review includes only six studies. Four studies were conducted using the same sample of CHR-P patients, although they investigated different primary outcomes in patients receiving either OT or placebo. One of the studies compared CHR-P individuals with healthy volunteers. The other two studies included psychosis patients, defining differently the term “early psychosis.” The study conducted by Cacciotti (15) included patients with a current or past diagnosis of schizophrenia spectrum disorder who were receiving treatment within 3 years of their diagnosis. Dagani et al. (16) examined schizophrenia subjects, within 1 year of their diagnosis, with a short-medium duration of illness.

In addition, only studies in English were included. Heterogeneity of the included studies, in terms of the outcome, and studies’ male predominance may have interfered with the findings on the efficacy of OT. Selection bias may be an issue for this review, concerning the subgroups of patients in the studies.

## 5. Conclusion

CHR-P individuals are defined by their negative and cognitive symptoms, attenuated psychotic symptoms, or by their short-lived remitting psychotic episodes. Established interventions aimed at CHR-P individuals may dependably enhance early detection and improve the disease course, limiting the first-episode onset chances and FEP severity, improving engagement with psychiatric services, and reducing comorbidity and duration of untreated illness (3). Therefore, future research should aim at this neglected group of patients, to help them benefit from the prevention and delay the FEP.

OT may affect the social cognition skills of people at prodromal and early stages of psychosis, but its effect on clinical symptoms is ambiguous. To conclude, because of the high level of heterogeneity, more original studies are needed to examine and clarify whether OT helps CHR-P and early psychosis populations.

## Author contributions

SB and NC: conceptualization, investigation, and methodology. SB: software, writing—original draft preparation, visualization, and project administration. SB, NC, and AP: validation. SB and AP: formal analysis and data curation. AP: resources and writing—review and editing. NC: supervision. All authors have read and agreed to the published version of the manuscript.

## Conflict of interest

The authors declare that the research was conducted in the absence of any commercial or financial relationships that could be construed as a potential conflict of interest.

## Publisher’s note

All claims expressed in this article are solely those of the authors and do not necessarily represent those of their affiliated organizations, or those of the publisher, the editors and the reviewers. Any product that may be evaluated in this article, or claim that may be made by its manufacturer, is not guaranteed or endorsed by the publisher.
