# Supplementary material for: Oxytocin as a treatment for high-risk psychosis or early stages of psychosis: a mini review
Source: Front Psychiatry. 2023 Aug 17;14:1232776. doi: 10.3389/fpsyt.2023.1232776 (PMC10470639; doi:10.3389/fpsyt.2023.1232776)
Supplement: Supplementary file 1 [file Data_Sheet_1.docx]

## Supplementary Material

## The different modes and doses of OT administration and its therapeutic role in psychiatric disorders

The therapeutic role of oxytocin in psychiatric disorders has been widely studied during the last decades, especially concerning its effects on social behavior in humans (1). The treatment of psychotic symptoms in schizophrenia patients using intravenously administered oxytocin was first reported by W.Bujanow in 1974 (2).

Oral administration of OT is followed by its decomposition by chymotrypsin in the gastrointestinal tract and quick absorption by mucous membranes. OT, then, enters the blood circulation, and reaches peripheral targets, without binding to plasma proteins (3). Orally introduced OT produces comparable effects to the intranasal route on humans’ social behavior on arousal and reward (4).

Intranasal administration of OT delivers the neuropeptide directly to the brain (5) (nose-to-brain drug route), although it is unclear how these particles cross the blood-brain barrier (BBB) through this route (6). It has been repeatedly observed to produce a decreased amygdala activation in a diversity of social cognitive and emotional situations (7) and it also influences the visual areas, as well as the fusiform gyrus (8,9). Quintana et al. demonstrated similar peripheral OT levels after intravenous and intranasal application. However, only the intranasal application of OT produced significant effects on the social cognition (10) and amygdala responses in fMRI (11).

A single dose of intranasal OT, ranging between 10 and 40 International Units (IU), is well tolerated, increases the answering score on a variety of tasks in social cognition (12), and ameliorates facial emotion recognition, along with several tasks investigating empathy, deception, and sarcasm in people diagnosed with schizophrenia (13).

It is argued that plasma OT levels are lower in people with schizophrenia with a negative correlation to psychotic symptoms (14). However, a review, by Rutigliano et al. (15) showed a lack of strong evidence for a meaningful difference between psychiatric patients (bipolar disorder, major depressive disorder, psychosis, autism spectrum disorders, anorexia nervosa, bulimia nervosa, obsessive-compulsive disorder) and healthy controls, regarding OT levels in plasma, saliva or cerebrospinal fluid. Thus, OT may not be used as an alleged biomarker. In contrast, a more recent study by Hernadez-Diaz Y., et al. stated that schizophrenia patients have elevated OT levels in cerebrospinal fluid but reduced levels in serum (16).

**Non-interventional studies not included in the review**

The two non-interventional studies, that of Bang et al. and Rubin et al. (17,18), that were not included in the review were conducted in Korea and USA respectively. The Korean study investigated the use of intranasal OT, although it studied the methylation of the oxytocin receptor gene (OXTR) on clinical and brain network connectivity phenotypes and its association with anhedonia-asociality in women at the early stages of psychosis. The other excluded study, by L. Rubin et al., examined the relationship between the endogenous levels of OT and arginine-vasopressin (AVP) with the severity of psychotic symptoms in acutely-ill unmedicated first-episode psychotic (FEP) patients. AVP levels, but not OT, associated with an increase in positive symptoms and a decrease in social cognition skills. These two studies enlightened that OT and its receptor (19) is an important neuropeptide concerning a specific psychotic population.

**Selection bias in oxytocin administration studies**

A significant limitation of the OT research is that most studies are, so far, conducted almost exclusively in males, rather than in both genders, (due to possible confounding factors related to the menstrual cycle). This fact questions the effects of OT’s applicability to females and, in turn, demonstrates a new clinical question, as OT’s effects present a possible medical treatment for various mental illnesses (focusing on psychosis and autism spectrum disorders).

One other study by Medved S. et al. showed that the residual, positive symptoms of schizophrenia in a newly diagnosed, early (one-week) post-partum, psychotic, young woman improved when OT was added to her treatment, through the mobilization of the dopaminergic-oxytocinergic pathway (20). This study clearly showed why it is important to include young women in the studying population (clinical high risk and early psychosis). Women are, currently, left out of most studied samples of schizophrenia patients, thus presenting an obvious selection bias.

**OT studies and implications in research**

It is well known that many studies on the OT effects on psychotic individuals use evidence from animal studies (21) or from healthy individuals (22) and are underpowered. Additionally, some positive results do not mirror the real effects of OT, whereas many real discoveries cannot be confirmed because of the lack of power of the replication studies, thus adding more confusion than benefit (23,24). It is highly recommended that studies with clearly defined outcomes be carefully designed and sufficiently powered. Moreover, publishing negative results and performing confirmatory studies of highly weighted, research-changing studies (such as the replication study by Declerc (25) on the Kosfeld experiment (26) is of great importance in pushing forward the research on OT administration.

The knowledge accumulated during all these years of research on OT’s brain effects (on the thalamus, parietal cortex, cerebellum, medial prefrontal cortex, striatum, ventral tegmental area, substantia nigra, fusiform gyrus, and amygdala) ought to be directed to the development of an effective OT formulation, suitable for patients during the prodromal and early stages of psychosis (27).

**References**

1. Peled-Avron L, Abu-Akel A, Shamay-Tsoory S. Exogenous effects of oxytocin in five psychiatric disorders: A systematic review, meta-analysis and a personalized approach through the lens of the social salience hypothesis. Neuroscience & Biobehavioral Reviews. 2020;

2. Bujanow W. Is oxytocin an anti-schizophrenic hormone? Canadian Psychiatric Association Journal. 1974;19(3):323–323.

3. Phelps C. The Anterior Pituitary and its Hormones. 2007;

4. Kou J, Lan C, Zhang Y, Wang Q, Zhou F, Zhao Z, et al. In the nose or on the tongue? Contrasting motivational effects of oral and intranasal oxytocin on arousal and reward during social processing. Transl Psychiatry. 2021 Feb 4;11(1):1–11.

5. Born J, Lange T, Kern W, McGregor GP, Bickel U, Fehm HL. Sniffing neuropeptides: a transnasal approach to the human brain. Nature neuroscience. 2002;5(6):514–6.

6. Magon N, Kalra S. The orgasmic history of oxytocin: Love, lust, and labor. Indian journal of endocrinology and metabolism. 2011;15(Suppl3):S156.

7. Wigton R, Radua J, Allen P, Averbeck B, Meyer-Lindenberg A, McGuire P, et al. Neurophysiological effects of acute oxytocin administration: systematic review and meta-analysis of placebo-controlled imaging studies. Journal of Psychiatry & Neuroscience. 2015;

8. Petrovic P, Kalisch R, Singer T, Dolan RJ. Oxytocin Attenuates Affective Evaluations of Conditioned Faces and Amygdala Activity. J Neurosci. 2008 Jun 25;28(26):6607–15.

9. Sauer C, Montag C, Wörner C, Kirsch P, Reuter M. Effects of a common variant in the CD38 gene on social processing in an oxytocin challenge study: possible links to autism. Neuropsychopharmacology. 2012;37(6):1474–82.

10. Quintana DS, Westlye LT, Rustan ØG, Tesli N, Poppy CL, Smevik H, et al. Low-dose oxytocin delivered intranasally with Breath Powered device affects social-cognitive behavior: a randomized four-way crossover trial with nasal cavity dimension assessment. Translational psychiatry. 2015;5(7):e602–e602.

11. Quintana DS, Westlye LT, Alnæs D, Rustan ØG, Kaufmann T, Smerud KT, et al. Low dose intranasal oxytocin delivered with Breath Powered device dampens amygdala response to emotional stimuli: A peripheral effect-controlled within-subjects randomized dose-response fMRI trial. Psychoneuroendocrinology. 2016;69:180–8.

12. Guastella AJ, MacLeod C. A critical review of the influence of oxytocin nasal spray on social cognition in humans: evidence and future directions. Horm Behav. 2012 Mar;61(3):410–8.

13. MacDonald E, Dadds MR, Brennan JL, Williams K, Levy F, Cauchi AJ. A review of safety, side-effects and subjective reactions to intranasal oxytocin in human research. Psychoneuroendocrinology. 2011 Sep;36(8):1114–26.

14. Cacciotti-Saija C, Langdon R, Ward PB, Hickie IB, Scott EM, Naismith SL, et al. A double-blind randomized controlled trial of oxytocin nasal spray and social cognition training for young people with early psychosis. Schizophrenia bulletin. 2015;41(2):483–93.

15. Rutigliano G, Rocchetti M, Paloyelis Y, Gilleen J, Sardella A, Cappucciati M, et al. Peripheral oxytocin and vasopressin: Biomarkers of psychiatric disorders? A comprehensive systematic review and preliminary meta-analysis. Psychiatry Res. 2016 Jul 30;241:207–20.

16. Hernández-Díaz Y, González-Castro TB, Tovilla-Zárate CA, López-Narváez ML, Genis-Mendoza AD, Castillo-Avila RG, et al. Oxytocin levels in individuals with schizophrenia are high in cerebrospinal fluid but low in serum: A systematic review and meta-analysis : Oxytocin and Schizophrenia. Metab Brain Dis. 2021 Dec;36(8):2415–24.

17. Bang M, Kang JI, Kim SJ, Park JY, Kim KR, Lee SY, et al. Reduced DNA methylation of the oxytocin receptor gene is associated with anhedonia-asociality in women with recent-onset schizophrenia and ultra-high risk for psychosis. Schizophrenia bulletin. 2019;45(6):1279–90.

18. Rubin LH, Carter CS, Bishop JR, Pournajafi-Nazarloo H, Harris MS, Hill SK, et al. Peripheral vasopressin but not oxytocin relates to severity of acute psychosis in women with acutely-ill untreated first-episode psychosis. Schizophrenia research. 2013;146(1–3):138–43.

19. Schiele MA, Thiel C, Kollert L, Fürst L, Putschin L, Kehle R, et al. Oxytocin Receptor Gene DNA Methylation: A Biomarker of Treatment Response in Obsessive-Compulsive Disorder? Psychother Psychosom. 2021;90(1):57–63.

20. Medved S, Bajs JanoviĆ M, Štimac Z, MihaljeviĆ-Peleš A. Add-on Oxytocin in the Treatment of Postpartum Acute Schizophrenia: A Case Report. J Psychiatr Pract. 2021 Jul 28;27(4):326–32.

21. Feifel D, Shilling PD, MacDonald K. A review of oxytocin’s effects on the positive, negative, and cognitive domains of schizophrenia. Biological psychiatry. 2016;79(3):222–33.

22. Bradley ER, Woolley JD. Oxytocin effects in schizophrenia: reconciling mixed findings and moving forward. Neuroscience & Biobehavioral Reviews. 2017;80:36–56.

23. Shamay-Tsoory S, Young LJ. Understanding the oxytocin system and its relevance to psychiatry. Biological psychiatry. 2016;79(3):150–2.

24. Walum H, Waldman ID, Young LJ. Statistical and methodological considerations for the interpretation of intranasal oxytocin studies. Biological psychiatry. 2016;79(3):251–7.

25. Declerck CH, Boone C, Pauwels L, Vogt B, Fehr E. A registered replication study on oxytocin and trust. Nature Human Behaviour. 2020;4(6):646–55.

26. Kosfeld M, Heinrichs M, Zak PJ, Fischbacher U, Fehr E. Oxytocin increases trust in humans. Nature. 2005 Jun 2;435(7042):673–6.

27. Davies C, Paloyelis Y, Rutigliano G, Cappucciati M, De Micheli A, Ramella-Cravaro V, et al. Oxytocin modulates hippocampal perfusion in people at clinical high risk for psychosis. Neuropsychopharmacology. 2019;44(7):1300–9.
